# Methionine Sulfoxide Reductases of Archaea

**DOI:** 10.3390/antiox7100124

**Published:** 2018-09-20

**Authors:** Julie A. Maupin-Furlow

**Affiliations:** Department of Microbiology and Cell Science, University of Florida, Gainesville, FL 32611-0700, USA; jmaupin@ufl.edu; Tel.: +1-352-392-4095

**Keywords:** archaea, methionine sulfoxide reductase, reactive oxygen species, ubiquitin-like modification, thiol relay systems

## Abstract

Methionine sulfoxide reductases are found in all domains of life and are important in reversing the oxidative damage of the free and protein forms of methionine, a sulfur containing amino acid particularly sensitive to reactive oxygen species (ROS). Archaea are microbes of a domain of life distinct from bacteria and eukaryotes. Archaea are well known for their ability to withstand harsh environmental conditions that range from habitats of high ROS, such as hypersaline lakes of intense ultraviolet (UV) radiation and desiccation, to hydrothermal vents of low concentrations of dissolved oxygen at high temperature. Recent evidence reveals the methionine sulfoxide reductases of archaea function not only in the reduction of methionine sulfoxide but also in the ubiquitin-like modification of protein targets during oxidative stress, an association that appears evolutionarily conserved in eukaryotes. Here is reviewed methionine sulfoxide reductases and their distribution and function in archaea.

## 1. Introduction

**Figure 1:**
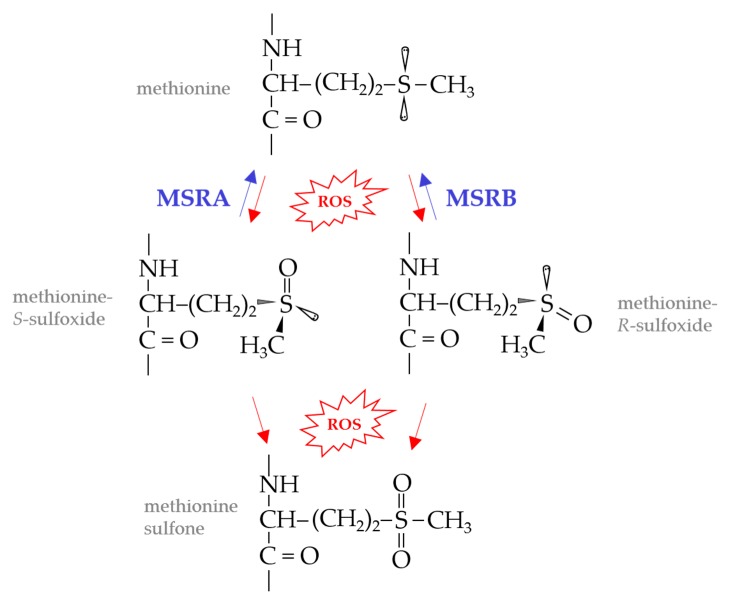
Methionine oxidation and repair. Methionine, whether in proteins or as a free amino acid, is readily oxidized to a mixture of methionine sulfoxide diasteromers by reactive oxygen species (ROS) and other oxidants. Of methionine sulfoxide reductase (MSR) enzymes, MSRA stereospecifically reduces methionine-*S*-sulfoxide, whereas MSRB is specific for the R-form of methionine sulfoxide. Methionine sulfoxides, if not repaired, may be further oxidized by ROS to produce methionine sulfone or radicals.

Reactive oxygen species (ROS), such as singlet oxygen (^1^O_2_), hydrogen peroxide (H_2_O_2_), superoxide anion (O^−^_2_) and hydroxyl radical (HO•), can cause widespread damage to cells. Proteins, amino acids, lipids, nucleic acids, and carbohydrates are generally susceptible to ROS damage [1,2]. The sulfur containing amino acids, methionine (Met) and cysteine (Cys), whether in protein or free form, are particularly sensitive to oxidation by ROS [3,4]. Met oxidation leads to the formation of diastereoisomers of methionine sulfoxide (Met-*S*-O and Met-*R*-O) which can be further oxidized to methionine sulfone (Figure 1). The accumulation of these oxidized methionine derivatives leads to protein carbonylation, aggregation and/or degradation. While methionine sulfone is irreversible, MetO can be repaired to Met by the action of methionine sulfoxide reductase (MSR) enzymes [5].

MSR enzymes are of structurally distinct families and substrate specificity. MSRA of the IPR036509 superfamily catalyzes the stereospecific reduction of Met-*S*-O in free and protein forms (Figure 1) and can reduce N-Ac-L-MetO, dimethylsulfoxide (DMSO), L-ethionine sulfoxide and sulindac [6,7,8]. MSRB of the IPR028427 family reduces free and protein forms of Met-*R*-O [9]. fRMSR of the GAF (cGMP-specific phosphodiesterases, adenylyl cyclases and FhlA)-like domain superfamily (IPR029016) reduces only the free form of Met-*R*-O [10,11,12]. MSRP of the molybdopterin-dependent sulfite oxidase family reduces free and protein forms of Met-*R*-O and Met-*S*-O [13,14] and can reduce dimethylsulfoxide (DMSO), trimethylamine-*N*-oxide (TMAO) and phenylmethyl sulfoxide in vitro [15]. Members of the molybdopterin-dependent DMSO reductase family, such as BisC [16,17], DmsA [18], TorZ/MSRZ [19] and BisZ [20], reduce free and/or protein forms of MetO in addition to other substrates such as biotin sulfoxide, nicotinamide-*N*-oxide, adenosine-*N*-oxide, DMSO and/or TMAO.

**Figure 2:**
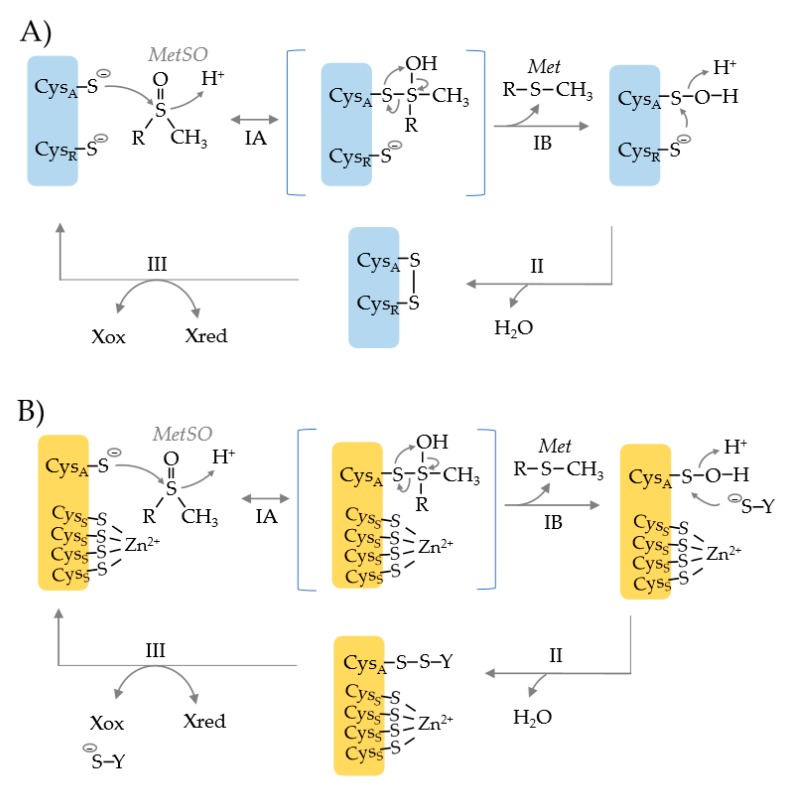
Catalytic mechanism of methionine sulfoxide reductases that use an active site cysteine nucleophile (Cys_A_) and a resolving cysteine (Cys_R_) (blue) or a single Cys_A_ active site (orange). (**A**) Cys_A_ attacks the S atom of the methionine sulfoxide (MetSO) substrate resulting in the formation of a tetrahedral transition state (step IA). The intermediate is rearranged to release the product methionine (Met) and form a Cys_A_ sulfenic acid (step IB). Cys_R_ attacks the S atom of the sulfenic acid, resulting in the release of water and the formation of an intradisulfide bond (Cys_A_-Cys_R_) (step II). The disulfide bond can be rearranged by other Cys residues (not shown), but ultimately must be reduced to regenerate the MSR enzyme (step III), where X represents a thiol relay system such as NAD(P)H > thioredoxin reductase (TrxR) > thioredoxin (Trx). (**B**) MSR enzymes that use a Cys_A_ nucleophile but rely upon a resolving agent (^−^S-Y), as exemplified by the *Methanothermobacter thermoautotrophicus* Zn^2+^-type MSRB (MTH711). Only the active site nucleophile (Cys_A_) is directly involved in catalysis. The four cysteine residues (Cys_S_) that coordinate the Zn^2+^ ion are used for structural integrity. The Cys_A_ sulfenic acid intermediate is resolved by formation of an interdisulfide bond with ^−^S-Y (step II) that is reduced by X_red_ (step III).

Common to MSRA, MSRB and fRMSR is the use of an active site cysteine (Cys_A_) to catalyze the nucleophilic attack of the S atom of the MetO substrate (Figure 2) [21]. This attack results in the formation of a tetrahedral transition state which is rearranged to release Met (the product) and form a Cys_A_ sulfenic acid. To resolve this inactive state, a resolving cysteine (Cys_R_) or reductant such as glutathione (YS^−^) serves as the nucleophile to attack the S atom of the Cys_A_ sulfenic acid (Figure 2, upper vs. lower). This attack releases water and forms a disulfide bond. In the Cys_R_ mechanism, an intradisulfide bond is formed between the Cys_A_ and Cys_R_ residues that can be rearranged by other Cys residues on the enzyme. If a separate thiol molecule is used to resolve the Cys_A_ sulfenic acid, an interdisulfide bond is formed. Ultimately, the inter- and intra-disulfide bonds are reduced by thiol relay systems such as nicotinamide adenine dinucleotide (phosphate) hydrogen [NAD(P)H]-dependent thioredoxin reductase (TrxR)/thioredoxin (Trx) or glutathione reductase (GR)/glutathione (GSH)/glutaredoxin (Grx) systems [21]. This reduction recycles the MSR enzyme back to an active state.

**Figure 3:**
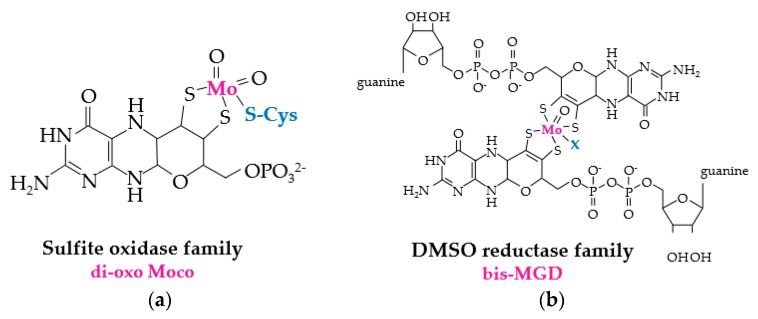
Molybdopterin prosthetic groups of MSR enzymes. (**a**) di-oxo Moco of sulfite oxidase family members such as MSRP (where Cys that coordinates the prosthetic group is represented in blue). (**b**) bis-MGD (molybdopterin guanine dinucleotide) prosthetic group of DMSO (dimethylsulfoxide) reductase family members such as BisC, BisZ, DmsA and TorZ/MSRZ which display MetO reductase activity. X (in blue) represents the additional ligand of bis-MGD which can be a serine, a cysteine, a selenocysteine, an aspartate or a hydroxide and/or water molecule.

The reduction of MetO by molybdopterin-dependent enzymes relies upon two distinct prosthetic groups (Figure 3). Enzymes of the DMSO reductase family use bis-MGD (molybdopterin guanine dinucleotide), a complex of Mo and two molybdopterin guanine dinucleotide (MGD) cofactors, for the redox-active prosthetic group, while members of the sulfide oxidase family coordinate a di-oxo form of the molybdenum cofactor (di-oxo Moco) as the redox-active center [22]. The source of electrons used for molybdopterin-dependent reduction of MetO varies. For example, the DMSO reductase family member BisC of *Rhodobacter sphaeroides* can use electrons directly from NAD(P)H [23], whereas *E. coli* BisC relies upon a protein-(SH)_2_ and flavoprotein relay system [24]. The sulfide oxidase family protein MSRP, which coordinates di-oxo Moco through a conserved Cys residue [15], resides in the periplasmic space of gram-negative bacteria [13]. MSRP reduces MetO using electrons from MSRQ, an integral b-type heme transmembrane protein of the NADPH oxidase family [13,14], which shuttles electrons from the quinone pool [13,14].

## 2. An Archaeal Perspective on Methionine Sulfoxide Repair

Archaea are microbes of a domain of life distinct from bacteria and eukaryotes [25]. Archaea are well known for their ability to withstand harsh environmental conditions that range from habitats of high ROS, such as hypersaline lakes with intense UV radiation and desiccation, to hydrothermal vents with low concentrations of dissolved oxygen at the high temperature [26]. Life evolved in an anaerobic world; thus, early archaea were relatively free from the damaging effects of ROS [27] and likely used a Wood–Ljungdahl pathway to generate energy and assimilate carbon (acetyl-CoA) from H_2_ and CO_2_ [28,29]. As oxygen levels increased over time, archaea and other microbes were challenged to develop mechanisms to repair and protect against ROS damage. Most archaea today, including strict anaerobes, have at least one antioxidant enzyme to protect and/or repair against ROS damage such as superoxide dismutase, catalase, peroxidiredoxin, superoxide reductase and/or MSR enzymes [30,31] (Table S1). The archaeal MSR enzymes are of particular interest in terms of evolutionary history [25,28,29] and how protein homeostasis is maintained in extreme habitats [26], as these enzymes are recently linked to ubiquitin-like protein modification pathways that are associated with oxidative stress and sulfur mobilization [32].

## 3. Archaeal Methionine Sulfoxide Reductase Homologs

The MSR homologs of archaea include: (i) MSRA and MSRB, which are prevalent in archaea but notably absent from most (hyper)thermophiles, (ii) fRMSR and MSRP, which are noted in archaea but are not common, and (iii) molybdopterin (MPT)/tungstopterin (WPT) oxidoreductase (OR) enzymes of the sulfite oxidase and DMSO reductase families (e.g., DMSO reductase, formate dehydrogenase, assimilatory nitrate reductase and formylmethanofuran dehydrogenase), which are widespread in archaea but are not known to function in MetO reduction [33,34,35,36] (Table S1).

**Figure 4:**
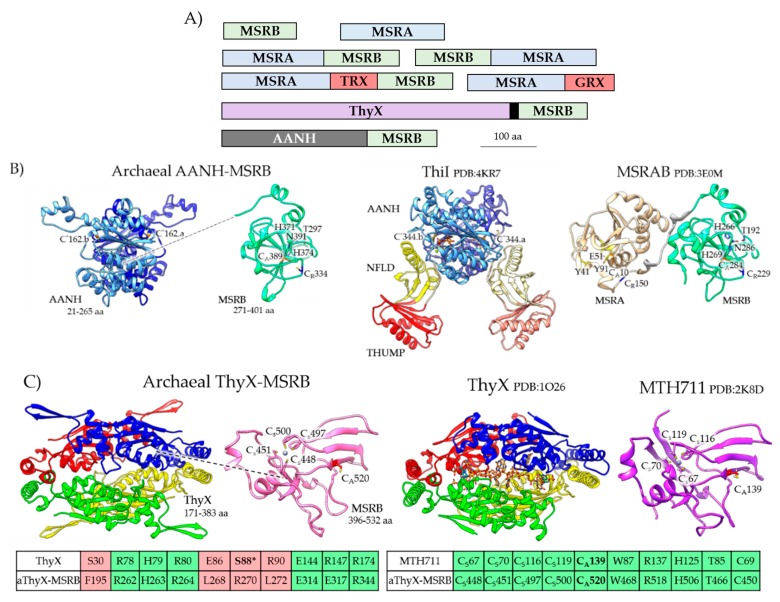
Archaeal MSRA/B stand-alone and fusion protein homologs. (**A**) Protein domain architecture of archaeal MSRA/B homologs. The black box indicates the coiled coil domain that links the ThyX and MSRB domains. (**B**) Archaeal AANH-MSRB (e.g., Uniprot: A0A2D6JNB0) has a predicted structural fold related to the adenylation (AANH) domain of tRNA sulfurtransferases such as *Thermotoga maritima* ThiI (Protein Data Bank or PDB: 4KR7) and the MSRB domain of *Streptococcus pneumoniae* MSRAB (PDB:3E0M). The active site cysteine nucleophiles of the AANH (Cys*162) and MsrB (Cys_A_389) domains as well as the resolving Cys residue of MsrB (Cys_R_334) are conserved. The N-terminal ferredoxin-like domain (NFLD) and RNA binding THUMP (thiouridine synthases, RNA methylases and pseudouridine synthases) domain of ThiI are not conserved. (**C**) Archaeal aThyX-MSRB (Uniprot A0A075GM99) has a predicted structural fold related to *Thermotoga maritima* flavin-dependent thymidylate synthase (ThyX, PDB: 1O26) and the Zn^2+^-binding MSRB of *Methanothermobacter thermautotrophicus* (MTH711, PDB: 2K8D). The ThyX flavin-binding site and MSRB Cys_A_ nucleophile and Cys_S_ Zn^2+^ coordinating residues are conserved; however, the serine residue (S88*) required for ThyX catalytic activity is not conserved in the aThyX-MSRB. TRX: thioredoxin domain; GRX: glutaredoxin domain.

The MSRA and MSRB homologs of archaea are stand-alone and fusion proteins (Figure 4A, Table S2). While most archaeal MSRA/B homologs are cytosolic, a subset have transmembrane spanning domains and signals for protein translocation via the general secretory (Sec) and/or twin arginine translocation (TAT) pathways. Of the archaeal fusion proteins, the MSRAB, MSRBA, MSRA-Trx-MSRB and MSRA-Grx share a general domain architecture with proteins of bacteria (e.g., MSRAB, MSRBA, and Trx-MSRAB) [37] and certain eukaryotes (e.g., *Entamoeba invadens* MSRAB, UniProt S0B0R4). The Trx/Grx domains likely facilitate a thiol relay to reduce MSRA/B, while the fusion of MSRA to MSRB may enhance the catalytic efficiency of MetO reduction [38]. One MSR homolog that appears unique to archaea is the fusion of MSRB to an N-terminal adenylation (AANH) domain (IPR020536) (Figure 4B). The AANH domain includes a conserved cysteine that is used by tRNA sulfurtransferases to catalyze the nucleophilic attack of ATP and form an adenylated tRNA intermediate during the transfer of sulfur to tRNA [39]. The archaeal AANH-MSRB homolog is missing the THUMP (thiouridine synthase, RNA methylase and pseudouridine synthase domain) domain used by the tRNA sulfurtransferases to bind tRNA [40]. Thus, while the archaeal MSRB-AANH is predicted to catalyze the reduction of MetO and the adenylation of a substrate (X), the identity of X has yet to be determined. In certain *Thaumarchaeota*, MSRB is fused to a ThyX-like domain (Figure 4C). ThyX is a flavin-dependent thymidylate synthase that uses reduced flavin to relay a methylene from a folate carrier to the deoxyuridine monophosphate (dUMP) acceptor to form deoxythymidine monophosphate (dTMP) [41]. While the archaeal ThyX-MSRB likely binds flavin and reduces MetO, the enzyme is not predicted synthesize dTMP as the Ser required for ThyX activity [42,43] is not conserved (Figure 4C). Thus, an unusual mechanism of electron transfer may occur in some archaeal MSR enzymes in which a flavin group, bound by a ThyX-like domain, may relay electrons for MSRB-mediated reduction of MetO.

## 4. Archaeal MSR Enzymes Characterized

The archaeal MSR enzymes that are purified and biochemically characterized are summarized below and in Table S3.

An fRMSR enzyme is characterized from the thermophilic archaeon *Thermoplasma acidophilum* (Ta) [44]. TafRMSR is the only archaeal MSR enzyme to date that can use an NADPH > Trx > TrxR system to reduce MetO in vitro [44]. Similarly to other fRMSR enzymes, TafRMSR reduces the free form of Met-*R*-O, not the protein MetO or Met-*S*-O forms [44]. TafRMSR has three cysteine residues (Cys15, Cys60 and Cys84). Of these, Cys60 and Cys84 are critical for MetO reductase activity, whereas, Cys15 is unimportant [44]. Cys84 forms the active site nucleophile (Cys_A_) [44]. Cys60, while important for activity, does not form a disulfide bond with Cys84 and, thus, is not considered a resolving cysteine (Cys_R_) [44]. TafRMSR is instead proposed to use a separate thiol to resolve the Cys_A_ active site. By contrast, bacterial and yeast fRMSR enzymes form intrachain disulfide bonds between catalytic and resolving cysteine residues (Cys_A_-Cys_R_) in the catalytic mechanism of MetO reduction [10,45,46,47].

MSRA/B homologs are not common among the hyperthermophiles; however, an MSRAB fusion is described from the hyperthermophilic archaeon *Thermococcus kodakarensis* (Tk). The Tk MSRAB enzyme reduces free and protein forms of MetO, with steroselectivity of the MSRA and MSRB domains as observed in bacteria and eukaryotes [48]. Tk MSRAB does not bind Zn^2+^ and is instead proposed to be acquired by horizontal gene transfer from bacteria [48]. Consistent with this evolutionary history, the Tk MSRAB is less active and less abundant at 85 °C (the optimal growth temperature) compared to temperatures below 80 °C where dissolved oxygen is higher [48]. Hydrothermal vent communities encounter drastic thermal gradients from the hot venting fluid and cold seawater; thus, MSRAB is thought to provide a selective advantage to *Thermococcus* species that prevail in vent ecosystems [49] where ROS damage may increase as temperatures drop.

MSRB enzymes that coordinate Zn^2+^ and require formation of an intramolecular thiol to regenerate the Cys_A_ active site are common in archaea and exemplified by MTH711 from the thermophilic methanogen *Methanobacterium thermoautotrophicus* [50]. Zn^2+^-containing MSRBs, such as MTH711, are apparent prototypes of the MSRB enzymes that lost the metal later in evolution [51]. Like other MSRBs, MTH711 can reduce free and protein forms of Met-*R*-O [50]. The Cys_A_ of MTH711 is directly involved in catalysis and can be reduced in vitro by dithiothreitol (DTT) or cysteine [50]. By contrast, the four cysteine residues (Cys_S_) that coordinate the Zn^2+^ ion are only required for structural integrity, and the two non-Zn^2+^-binding Cys residues that reside outside the catalytic center are not needed for catalysis [50]. Thus, the Cys_A_ sulfenic acid intermediate is proposed to be resolved by a thiol that is distinct from the MSRB enzyme [50,52].

Stand-alone MSRA and Zn^2+^-type MSRB enzymes are recently characterized from the halophilic archaeon *Haloferax volcanii*. These MSR enzymes reduce the peptide mimic, dabsyl-Met-(R/S)-O, with DTT serving as the reductant [32]. Futher analysis of MSRA reveals it requires a conserved active site nucleophile (Cys_A_, Cys13) and an invariant glutamate (Glu56, presumed to bind the MetO oxygen atom) for its activity [32]. The MetO reductase activity of MSRA is inhibited by DMSO [32], a competitive active site inhibitor based on analogy to yeast MSRA which reduces DMSO to DMS (dimethylsulfide) [53,54]. Surprisingly, DMSO stimulates an MSRA-dependent ubiquitin-like protein (Ubl) modification system in this archaeon as described below [32].

## 5. MSRA and Its Function in Ubiquitin-Like Protein Modification

In addition to its role as a MetO reductase, the *H. volcanii* MSRA has an apparent ‘moonlighting’ function in ubiquitin-like (Ubl) modification [32]. Archaea mediate Ubl modification by a mechanism that is related to eukaryotic ubiquitination [55,56]. An E1-like enzyme adenylates the Ubl and forms an E1~Ubl thioester intermediate prior to Ubl modification [57,58]. Most archaea are missing homologs of the classical E2 ubiquitin-conjugating and E3 ubiquitin-ligase enzymes of eukaryotic ubiquitination [56]. Thus, the latter stages of archaeal Ubl modification are unclear. In the presence of DMSO, an inhibitor of MetO reductase activity, MSRA stimulates the E1-dependent Ubl modification of target proteins [32] and is itself a target of Ubl modification [59]. This reaction is unaffected by (excess or limiting) DTT suggesting the MSRA mechanism occurs independent of its oxidase or reductase activities [32]. Further studies are needed to clarify the precise role of MSRA in this process.

## 6. MSRA/B Regulation in Archaea

Archaeal MSRA/B enzymes are regulated at the transcript, protein, and post-translational levels (Table S4). In *Halobacterium salinarum* (known for its purple membrane), the transcript levels of MSRA and MSRB are up during conditions of severe oxidative stress [60,61]. The *H. salinarum msrB* is one of the core genes of the RosR (reactive oxygen species transcriptional regulator) regulon that includes superoxide dismutase, Trx-like, and other related genes [60]. MSRA transcript levels are also found to be up in *Sulfolobus solfataricus* and *Sulfolobus acidocaldarius* (crenarchaeota from acidic hot springs) after UV irradiation [62], a condition known to generate ROS [63]. Likewise, MSRAB abundance is up at the protein level after exposure of *T. kodakarensis* to saturating oxygen, low temperature or high salt [48,64]. As discussed earlier, oxidative stress is associated with temperature downshifts from 80 °C due to the increase in dissolved oxygen concentration. Hyposaline and hypersaline conditions stimulate antioxidant responses in algae and plants [65] and appear to do so in archaea based on the Tk MSRAB response [64]. The MSRA and MSRB of *H. volcanii* are Ubl modified (sampylated) in the presence of the mild oxidant DMSO [59]. DMSO exposure reduces the level of unmodified MSRA and increases the level of Ubl-modified MSRA [32]. Thus, Ubl modification appears to target MSRA for proteolysis and may serve to autoregulate the oxidative stress response of this haloarchaeon.

## 7. Protein Disulfide Relay Systems of Archaea

**Figure 5:**
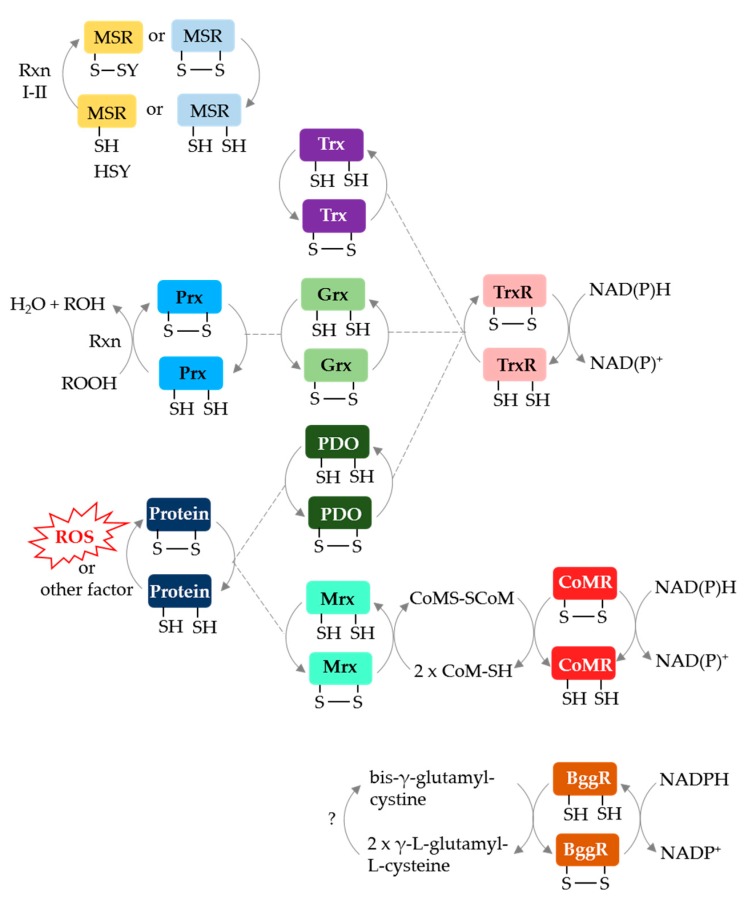
Archaeal protein disulfide relay systems which may provide reductant for MetO reduction by MSR enzymes that use a Cys_A_ mechanism. See text for details. Prx: peroxiredoxin; PDO: protein disulfide oxidoreductase; NAD(P)H: nicotinamide adenine dinucleotide (phosphate) hydrogen; CoMR: coenzyme M disulfide reductase; BggR: bis-γ-glutamylcystine reductase; CoMS-SCoM: coenzyme M disulfide.

Archaeal protein disulfide relay systems may provide the reductant for MetO reduction by MSR enzymes that use a Cys_A_ nucleophile. Archaeal NAD(P)H-dependent protein disulfide oxidoreductase relay systems which could serve this role include: (i) Trx or Grx/TrxR [66,67], (ii) protein disulfide oxidoreductase (PDO)/TrxR [68], (iii) methanoredoxin (Mrx)/coenzyme M disulfide reductase (CoMR) [69,70] and (iv) bis-γ-glutamylcystine reductase (BggR) [71,72] (Figure 5). An archaeal F420-dependent TrxR is also described [73]. Of these disulfide relay systems, Grx/TrxR can resolve the disulfide bond of peroxiredoxins (Prx), such as the alkyl hydroperoxide reductase AhpC [74]. Furthermore, PDO/TrxR and Mrx/CoMR can reduce the disulfide bonds of oxidized proteins [68,70]. While these systems are not yet linked to archaeal MSR enzymes, TafRMSR can use an NADPH-dependent *E. coli* Trx/human TrxR system to reduce MetO [44], suggesting that a native Trx/TrxR may resolve the Cys_A_ active site of TafRMSR. Trx and Grx domains are fused to MSRA/B in some archaea (see above) providing further evidence for disulfide relay systems.

## 8. Physiological Roles of Archaeal MSR Enzymes

In general, loss of MSR activity can reduce cell viability, increase ROS production, and promote protein carbonylation [75]. Archaea are no exception. *H. volcanii ΔmsrA* (when compared to wild type, *ΔmsrB* and *ΔmsrA msrA*+) has a pronounced decrease in the abundance of Ubl modified proteins that form after cells are exposed to the mild oxidant DMSO; subsequent transfer of these cells to severe oxidative stress (hypochlorite or H_2_O_2_) results in reduced cell viability [32]. Thus, the archaeal MSRA appears important in responses to oxidative stress that are physiologically distinct from MSRB [32].

## 9. Conclusions and Future Perspectives

Archaeal MSR enzymes are full of surprises. The dearth of MSRA/B homologs in hyperthermophiles and low temperature preference of the *Thermococcus* MSRAB suggest that Met oxidation may not be a limiting factor for extremophiles that live at high temperature and pressure. Alternatively, these archaea use an MSR enzyme that has yet to be discovered. The finding that TafRMSR and MSRB-type MTH711 use an active site Cys_A_ that is reduced by an external thiol and not a Cys_R_ suggests that archaea use a streamlined mechanism for resolving the active site. The most recent finding—that *H. volcanii* MSRA has a moonlighting function in Ubl modification—is also surprising and helped guide the insight that MSRA competes for capturing ubiquitin and promotes ubiquitination in mammals [76]. Whether archaeal MSR enzymes can extend the lifespan of an organism remains to be determined. Expression of fRMSR enzymes lost during evolution can lead to an increased lifespan in animals [77]. Likewise, expression of MSRA, together with DMSO, can also extend an organism’s life [78,79]. Thus, archaeal MSR enzymes, including those yet to be discovered, may hold a key to the fountain of youth.

## Supplementary Materials

The following are available online at http://www.mdpi.com/2076-3921/7/10/124/s1, Table S1: Phylogenetic distribution of methionine sulfoxide reductase homologs compared to 20S proteasome, superoxide dismutase and other oxidative stress response homologs. Table S2: Domain architecture of MSRA- and MSRB-type methionine sulfoxide reductase homologs among Archaea. Table S3: Archaeal methionine sulfoxide reductases biochemically and/or structurally characterized. Table S4: Archaeal methionine sulfoxide reductases regulated at the protein and/or transcript level.
