# Supplementary material for: Methionine Sulfoxide Reductases of Archaea
Source: Antioxidants (Basel). 2018 Sep 20;7(10):124. doi: 10.3390/antiox7100124 (PMC6211008; doi:10.3390/antiox7100124)
Supplement: Supplementary file 1 [file antioxidants-07-00124-s001.pdf]

Supplemental information

Table S1. Phylogenetic distribution of methionine sulfoxide reductase homologs compared to 20S proteasome, superoxide dismutase and other oxidative stress response homologs.

Phylogenetic classification	20S α-type	MsrA	MrsB	MsrP	fRMsr	MSR -MPT	MPT OR	MSR +MPT	OsmC/ Ohr	AhpC -type	Fe/Mn SOD	Ni SOD	Cu/Zn SOD
Bacteria	1854	29628	24688	5180	4990	+	88528	+	46232	44713	25113	1815	8763
Eukarya	12755	2542	2693	1	560	+	1886	+	511	5620	4306	34	6821
Archaea	1513	959	846	2	77	+	2441	+	1129	2442	866	124	25
Asgard	17	12	8	-	2	+	20	+	29	19	1	4	-
Heimdallarchaeota	4	4	2	-	2	+	7	+	6	4	-	4	-
Lokiarchaeota	5	3	2	-	-	+	7	+	7	3	-	-	-
Odinarchaeota	2	1	1	-	-	+	1	+	1	1	1	-	-
Thorarchaeota	6	4	3	-	-	+	5	+	15	11	-	-	-
DPANN	134	47	53	-	2	+	6	+	3	79	74	3	-
Aenigmarchaeota	10	-	-	-	-	-	-	-	-	6	7	-	-
Diapherotrites	7	1	1	-	-	+	1	+	-	4	2	-	-
Micrarchaeota	21	5	4	-	-	+	-	+	-	12	12	-	-
Nanohaloarchaeota	6	14	16	-	-	+	-	+	2	2	8	-	-
Pacearchaeota	44	16	21	-	1	+	-	+	-	19	27	-	-
Parvarchaeota	4	2	1	-	-	+	1	+	-	4	2	-	-
Woesearchaeota	36	9	10	-	1	+	4	+	1	25	12	3	-
Nanoarchaeota	6	-	-	-	-	-	-	-	-	7	4	-	-
Euryarchaeota	1016	766	656	2	54	+	1982	+	920	1816	567	113	23
Archaeoglobi	12	-	-	-	-	-	29	+	3	6	1	-	-
Hadesarchaea (70 °C)	6	-	-	-	-	-	3	+	2	2	2	-	-
Halobacteria	443	342	258	-	-	+	713	+	427	664	331	-	-
Methanobacteria	49	41	41	-	2	+	159	+	-	28	24	-	-
Methanococci	19	7	1	-	-	+	78	+	11	17	-	-	-
Methanomicrobia	142	141	119	-	1	+	494	+	72	268	94	-	22
Methanonatronarchaeia	2	2	2	-	-	+	3	+	-	2	-	-	-
Methanopyri	1	-	-	-	-	-	5	+	1	-	-	-	-

Theionarchaea	3	-	-	-	-	-	6	+	1	3	-	-	-
Thermococci	38	5	5	-	-	+	95	+	84	92	-	-	-
Palaeococcus	1	-	-	-	-	-	2	+	2	1	-	-	-
Pyrococcus	8	-	-	-	-	-	18	+	20	13	-	-	-
Thermococcus	28	4	4	-	-	+	73	+	59	75	-	-	-
unclassified	1	1	1	-	-	+	2	+	3	3	-	-	-
Thermoplasmata	53	40	33	-	17	+	29	+	56	132	26	1	-
Euryarchaeota_environment	6	9	6	-	-	+	15	+	2	15	5	1	-
Euryarchaeota_unclassified	242	179	191	2	34	+	356	+	261	587	84	111	1
<u>TACK</u>	265	91	86	-	13	+	319	+	102	439	180	3	2
Bathyarchaeota	29	5	2	-	-	+	34	+	24	10	4	-	-
Korarchaeota	4	1	-	-	1	+	1	+	4	5	1	-	-
Crenarchaeota	102	20	5	-	-	+	263	+	57	266	72	-	-
Acidilobales	9	-	-	-	-	-	15	+	1	24	8	-	-
Desulfurococcales	22	-	-	-	-	-	35	+	11	30	3	-	-
Fervidicoccales	3	-	-	-	-	-	4	+	-	4	-	-	-
Sulfolobales	28	15	-	-	-	+	114	+	37	121	29	-	-
Thermoproteales	33	-	-	-	-	-	93	+	3	70	25	-	-
Crenarchaeota_unclassified	7	5	5	-	1	+	2	+	5	17	7	-	-
Thaumarchaeota	130	65	79	-	-	+	19	+	17	158	103	3	2
Archaea_environment	19	12	12	-	3	+	90	+	4	17	8	-	-
Archaea_unclassified	62	31	31	-	3	+	24	+	71	72	36	1	-

[1]Phylogenetic distribution of archaeal homologs of: 20S proteasome α-type subunits (IPR023332), MsrA (IPR036509) (excludes type III ribulose bisphosphate carboxylase homologs of IPR017712 originally misannotated as MsrA [1]), MsrB (IPR0025790), MsrP/Q (IPR022867/IPR022837), fRMsr, free MetO reductase conserved site (IPR000614) with archaeal homologs based on at least 30 % amino acid sequence identity by BlastP UniProt to *Thermoplasma acidophilum* fRMsr (Ta0848), MPT OR, molybdopterin oxidoreductases such as BisC (IPR006656); peroxiredoxins of the OsmC/Ohr (IPR003718) and AhpC (IPR024706) families; superoxide dismutase of the Fe/Mn SOD (IPR001189), Ni SOD (IPR014123) and Cu/Zn SOD (IPR036423) types. -, not detected by http://www.ebi.ac.uk/interpro/ on August 6, 2018. (Hyper)thermophiles are indicated by red lettering with unclassified and environmental samples excluded from this latter analysis. Presence (green) or absence (pink) of a representative MSR homolog (MsrA, MsrB, fRMsr) excluding (MSR - MPT) or including (MSR + MPT) BisC-like MPT oxidoreductases is also indicated. Within the GAF-like domain superfamily, the conserved active site Cys_A motif of fRMsr was used to distinguish fRMsr homologs from signaling proteins such as cGMP-specific phosphodiesterases, adenylyl cyclases and guanylyl cyclases [2].

Table S2. Domain architecture of MsrA- and MsrB-type methionine sulfoxide reductase homologs among Archaea[1].

(Super)phyla	MsrA (single domain)	MsrB (single domain)	MsrA-MsrB	MsrB-MsrA	MsrA-Trx-MsrB	AANH-MsrB	ThyX-MsrB	MsrA-Grx
TACK	89	71	-	-	-	-	2	-
Asgard	10	7	-	2	-	-	-	-
Euryarchaeota	668	536	78	10	2	61	-	1
DPANN_Woesearchaeota	6	6	6	-	-	1	-	-
DPANN_Pacearchaeota	13	17	4	2	-	-	-	-
DPANN_Parvarchaeum	2	1	-	-	-	-	-	-
DPANN_Nanoarchaeota	-	-	-	-	-	-	-	-
DPANN_Nanohaloarchaeota	10	15	-	-	-	-	-	-
DPANN_Micrarchaeota	5	4	-	-	-	-	-	-
DPANN_Diapherotrites	1	1	-	-	-	-	-	-
Archaea_Unclassified	36	37	8	-	-	-	-	-
Total:	840	695	96	14	2	62	2	1
(SignalP, TAT or TM)[2]	15	10	22	2	2	1	-	-

[1]Methioinine sulfoxide reductase MsrA-type (IPR036509) and MsrB-type (IPR002579) homologs are found as single domain proteins or fused in tandem or with alternative domains including: ThyX (thymidylate synthase, IPR003669), AANH (adenylation) domain of the QueC-type (queosine biosynthesis protein QueC, IPR018317), Grx (glutaredoxin, IPR002109) or Trx (thioredoxin-like, IPR036249) domains. [2]A subset of MsrA- and MsrB-type homologs have predicted TM (transmembrane spanning helix based on http://www.cbs.dtu.dk/services/TMHMM/), TAT (twin arginine translocation (TAT) motifs based on http://signalfind.org/tatfind.html) and/or SignalP (signal sequence) motifs for general (Sec) translocation based on http://www.cbs.dtu.dk/services/SignalP/).

Table S3. Archaeal methionine sulfoxide reductases biochemically and/or structurally characterized.

Organism	MSR-type (locus tag)	Substrate	Reductant	Active site	Ubl site	PDB	Ref.
Thermococcus kodakarensis	MsrAB (TK0819)	MetO	DTT	*C17 (A)*<br>*C20 (R)?*<br>*C278 (R)?*<br>*C283 (R)?*<br>*C311 (A)*	-	-	[3]
	MsrA' (TK0819)	Met-*S*-O, dabsyl-Met-*S*-O	DTT				
	'MsrB (TK0819)	Met-*R*-O, dabsyl-Met-*R*-O	DTT, cysteine				
Methanothermobacter thermoautotrophicus	MsrB (MTH711)	Met-*R*-O, Lys-Ile-Phe-MetO-Lys, H_2O_2-oxidized calmodulin	DTT, cysteine	C64 (Zn)<br>C67 (Zn)<br>C113 (Zn)<br>C116 (Zn)<br>C139 (A)	-	2K8D	[4, 5]
Thermoplasma acidophilum	fRMsr (Ta0848)	Met-*R*-O	NADPH > *E. coli* Trx > human Trx reductase	C84 (A)<br>C60 (C)	-	4MMN<br>4MN7	[6]
Haloferax volcanii	MsrA (HVO_A0230)	dabsyl-Met-(*R*/*S*)-O	DTT	C13 (A)<br>*C16 (R)?*<br>*C48 (R)?*<br>*C162 (R)?*	K108, K169, K172, K180, K182	-	[7, 8]
	MsrB (HVO_2234)	dabsyl-Met-(*R*/*S*)-O	DTT	*C49 (Zn)*<br>*C52 (Zn)*<br>*C97 (Zn)*<br>*C100 (Zn)*<br>*C120 (A)*	K117	-	

-, not determined. PDB, Protein Data Bank accession number. Cysteine nucleophile (A), resolving (R), Zn^{2+} binding (Zn) or critical (C) residues are indicated with predicted in italics. Ubl, ubiquitin-like modification site. MsrA' and 'MsrB, are domains isolated from MsrAB. Trx, thioredoxin.

Table S4. Archaeal methionine sulfoxide reductases regulated at the protein and/or transcript level.

Organism	MSR-type (locus tag)	Condition	Effect	Ref.
Thermococcus kodakarensis	MsrAB (TK0819)	Low temperature	Protein level – up	[3]
		High salt	Protein level – up	[9]
		Oxidative stress (saturating oxygen)	Protein level – up	[9]
Sulfolobus solfataricus	MsrA (SSO1503)	UV irradiation	Transcript level – up	[10]
Sulfolobus acidocaldarius	MsrA (Saci_1170)	UV irradiation	Transcript level – up	[10]
Halobacterium salinarum	MsrB (VNG1404G)	Oxidative stress (paraquat, H_2O_2)	Transcript level – up (member of RosR regulon core)	[11]
Halobacterium salinarum	MsrA (VNG1180G)	Oxidative stress (paraquat, H_2O_2)	Transcript level – up	[12]
Haloferax volcanii	MsrA (HVO_A0230)	Mild oxidant (DMSO)	Protein level: Ubiquitin-like modified form – up Unmodified form – down	[8]

References (for supplemental information)

1. Tabita F.R.; Hanson T.E.; Li H.; Satagopan S.; Singh J.; Chan S. Function, structure, and evolution of the RubisCO-like proteins and their RubisCO homologs. *Microbiol Mol Biol Rev*. **2007**;*71*(4):576-99.
2. Lin Z.; Johnson L.C.; Weissbach H.; Brot N.; Lively M.O.; Lowther W.T. Free methionine-(R)-sulfoxide reductase from *Escherichia coli* reveals a new GAF domain function. *Proc Natl Acad Sci U S A*. **2007**;*104*(23):9597-602.
3. Fukushima E.; Shinka Y.; Fukui T.; Atomi H.; Imanaka T. Methionine sulfoxide reductase from the hyperthermophilic archaeon *Thermococcus kodakaraensis*, an enzyme designed to function at suboptimal growth temperatures. *J Bacteriol*. **2007**;*189*(19):7134-44.
4. Carella M.; Becher J.; Ohlenschläger O.; Ramachandran R.; Gührs K.H.; Wellenreuther G.; Meyer-Klaucke W.; Heinemann S.H.; Görlach M. Structure-function relationship in an archaebacterial methionine sulphoxide reductase B. *Mol Microbiol*. **2011**;*79*(2):342-58.
5. Carella M.; Ohlenschläger O.; Ramachandran R.; Görlach M. ^{1}H, ^{13}C and ^{15}N resonance assignment of a zinc-binding methionine sulfoxide reductase type-B from the thermophilic archeabacterium *Methanothermobacter thermoautotrophicus*. *Biomol NMR Assign*. **2010**;*4*(1):93-5.
6. Kim H.S.; Kwak G.H.; Lee K.; Jo C.H.; Hwang K.Y.; Kim H.Y. Structural and biochemical analysis of a type II free methionine-R-sulfoxide reductase from *Thermoplasma acidophilum*. *Arch Biochem Biophys*. **2014**;*560*:10-9.
7. Dantuluri S.; Wu Y.; Hepowit N.L.; Chen H.; Chen S.; Maupin-Furlow J.A. Proteome targets of ubiquitin-like samp1ylation are associated with sulfur metabolism and oxidative stress in *Haloferax volcanii*. *Proteomics*. **2016**;*16*(7):1100-10.
8. Fu X.; Adams Z.; Liu R.; Hepowit N.L.; Wu Y.; Bowmann C.F.; Moskovitz J.; Maupin-Furlow J.A. Methionine sulfoxide reductase A (MsrA) and its function in ubiquitin-like protein modification in Archaea. *MBio*. **2017**;*8*(5):e01169-17.
9. Jia B.; Liu J.; Van Duyet L.; Sun Y.; Xuan Y.H.; Cheong G.W. Proteome profiling of heat, oxidative, and salt stress responses in *Thermococcus kodakarensis* KOD1. *Front Microbiol*. **2015**;*6*:605.
10. Gotz D.; Paytubi S.; Munro S.; Lundgren M.; Bernander R.; White M.F. Responses of hyperthermophilic crenarchaea to UV irradiation. *Genome Biol*. **2007**;*8*(10):R220.
11. Sharma K.; Gillum N.; Boyd J.L.; Schmid A. The RosR transcription factor is required for gene expression dynamics in response to extreme oxidative stress in a hypersaline-adapted archaeon. *BMC Genomics*. **2012**;*13*:351.
12. Kaur A.; Van P.T.; Busch C.R.; Robinson C.K.; Pan M.; Pang W.L.; Reiss D.J.; DiRuggiero J.; Baliga N.S. Coordination of frontline defense mechanisms under severe oxidative stress. *Mol Syst Biol*. **2010**;*6*:393.
